# A new function for thermal phase transition-based polymer actuators: autonomous motion on a surface of constant temperature†
†Electronic supplementary information (ESI) available: Additional figures, tables, discussions and experimental information. See DOI: 10.1039/c7sc01792h



**DOI:** 10.1039/c7sc01792h

**Published:** 2017-07-03

**Authors:** Feijie Ge, Yue Zhao

**Affiliations:** a Département de Chimie , Université de Sherbrooke , Sherbrooke , Québec J1K 2R1 , Canada . Email: yue.zhao@usherbrooke.ca

## Abstract

It is very challenging to make materials capable of autonomous oscillation known in many living systems (such as the heartbeat). Herein, we describe an approach to creating a thermo-mechano-thermal feedback loop for thermal phase transition-based polymer actuators, which leads to hour-long, autonomous motion on a substrate surface of constant temperature. We investigated the variables that determine the amplitude and period of the motion, and demonstrated exemplary physical work powered by direct thermomechanical energy conversion. Such continuous motion of a solid polymer driven by thermal energy without the need for temperature up/down switching is unprecedented, and the validated feedback loop can be implemented into other thermal phase transition-based polymer actuators.

## Abstract 2

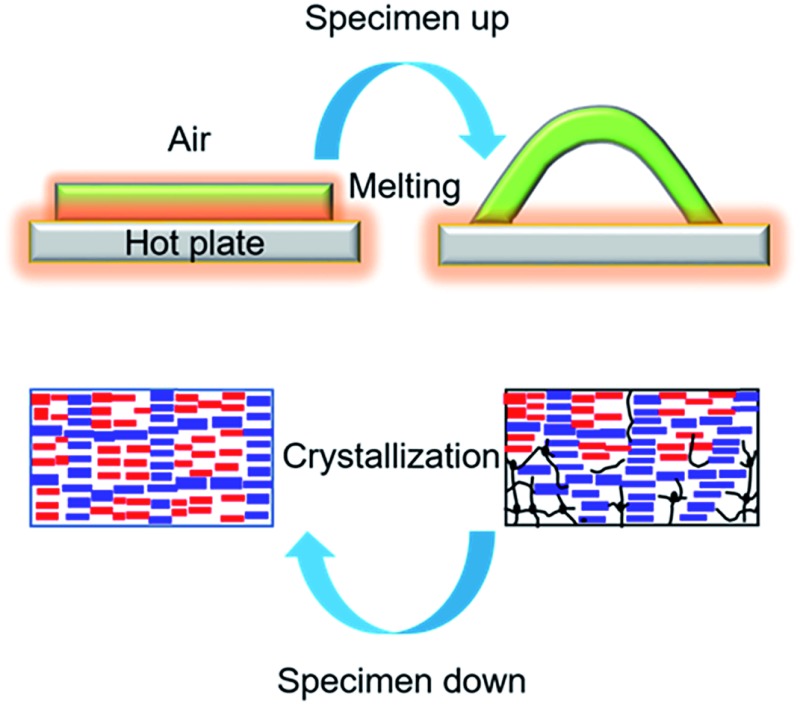
A thermo-mechano-thermal feedback loop allows solid polymer actuators to undergo hour-long, autonomous motion on a substrate surface of constant temperature.

## Introduction

Polymers that are responsive to diverse stimuli have been continuously exploited for application as soft actuators in the past few decades.^1^ But a major challenge arises when it comes to autonomous actuators that, like in living systems (*e.g.* the heartbeat), can undergo continuous, self-sustained oscillating motion with no need for on/off switching of external stimuli, *i.e.*, without external intervention. The rare systems capable of autonomous actuation are mainly stimuli-responsive polymer brushes^2^ and hydrogels under the effect of an oscillating reaction^3^ or a water-gradient,^4^ and photoresponsive polymers based on the photothermal effect^5^ or photoisomerization reactions.^6^ In the latter case, liquid crystal networks (LCNs) bearing azobenzene moieties are the most developed, for which the reversible *trans*–*cis* photoisomerization of azobenzene is at the origin of either rotation under synchronized irradiation with two separate beams of UV and visible light,^7^ oscillation of a cantilever under one focused light beam^8^ or chaotic oscillation driven by sunlight.^9^ Other examples of autonomous actuators are powered by triggered depolymerization reactions^10^ or catalytic decomposition.^11^ Of particular note is a hydrogel-based microstructure developed by Aizenberg’s group,^12^ which is capable of self-sustained oscillation between two liquid layers, driven by a chemo-mechano-chemical feedback loop. The key to achieving autonomous actuation under unchanged or “constant” stimulation is a feedback loop. Besides the aforementioned systems, thermally fuelling a solid polymer actuator for continuous motion has not been realised thus far. Herein, we demonstrate a strategy for creating a thermo-mechano-thermal feedback loop that allows a solid polymer to undergo continuous, self-powered actuation with the only requirement being exposure to a substrate surface of constant temperature. We also investigated the factors that determine the amplitude and period of the thermally driven oscillating motion.

**Fig. 1:**
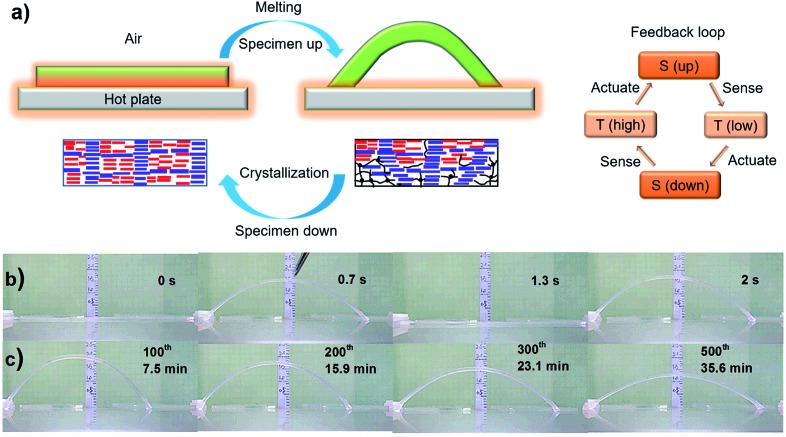
Autonomous actuation. (a) Schematic for the melting-induced superficial contraction making the specimen arch up and the crystallization-induced superficial extension bringing the specimen down, which goes on in a continuous and self-powered manner due to a thermo-mechano-thermal feedback loop (S: sample, *T*: temperature); the blue arrays and red arrays represent polymer crystallites with high *T*_m_ and low *T*_m_, respectively, while the black lines and dots represent the amorphous polymer chains and crosslinks, respectively. (b) Snapshots showing the first two jumps of an EVA specimen (100% elongation, 65 × 3 × 0.6 mm^3^) placed on the surface of a steel plate at *T*_surf_ = 84 °C. (c) Snapshots showing the actuation amplitude of the same specimen after hundreds of jump/descent movements (the number of cycles and the corresponding actuation times are indicated).

Our envisioned thermo-mechano-thermal feedback loop can be depicted as follows (Fig. 1a). When the lower side of a polymer strip with oriented chains touches the substrate surface, should a temperature gradient be quickly established, the polymer may undergo a phase transition (*e.g.*, melting) mainly in the lower surface region to give rise to an unbalanced longitudinal contractile force that pushes the middle section of the strip upward to form an arch. Once in the air, as the lower side of the strip cools down, the reverse phase transition occurs, resulting in an extensional force that brings the deformed strip back to the flat shape and, by doing so, makes the lower side touch the surface again to renew the motion cycle. To validate this design and the related mechanism, we chose to use a crosslinked semicrystalline random copolymer poly(ethylene-*co*-vinyl acetate) (EVA) that, as reported by Lendlein and coworkers,^13,14^ is a thermal polymer actuator displaying a two-way shape memory, or temperature-memory, effect. The melting temperature *T*_m_ of EVA spans a wide range of about 40 °C, between *T*_m,low_ and *T*_m,high_. After elongating a specimen in the melt (*T* > *T*_m,high_), cooling it under strain induces crystallization of the oriented polymer chains and allows the elongated shape to be frozen at room temperature below *T*_m,low_. After this programming phase, by heating the elongated specimen to a temperature T* within the melting range (*T*_m,low_ < T* < *T*_m,high_), crystallites with *T*_m_ < T* melt in the actuation domains, while those with *T*_m_ > T* remain and form a rigid skeleton, resulting in a contraction force that shrinks the specimen along the elongation direction. On subsequent cooling, oriented chain segments in the actuation domains recrystallize under the constraints of the crystalline skeleton, giving rise to an expansion force that elongates the specimen to the initial shape. Therefore, reversible thermal actuation (shrinkage/extension) is achieved by switching the temperature up and down. In a previous study,^15^ we transformed this thermal actuator onto an optical actuator by loading a small amount of gold nanoparticles (AuNPs) in EVA, and used the photothermal effect arising from the surface plasmon resonance (SPR) of the nanofiller to realise the on–off switching of temperature through the on–off switching of light. It was found that with one side of the specimen exposed to light, a temperature gradient could be formed due to attenuation of light absorption along the thickness direction, leading to reversible bending/unbending upon light on/off respectively. Inspired by this finding, it could be foreseen that when a sufficiently thick specimen is in contact with a hot substrate surface, a temperature gradient could be formed due to heat diffusion along the thickness direction, which leads to a superficial melting-induced contractile force that pushes the specimen up and, once cooled in the air, yields a crystallization-induced extensional force that flattens the specimen on the substrate surface to reactivate the motion cycle. Therefore, EVA was utilised in the present study to investigate the thermally driven autonomous motion under the envisioned thermo-mechano-thermal feedback loop. Before discussing the results, it should be emphasized that the reversible actuation mechanism of EVA was reported in ref. 13, while the present study is about a mechanism of autonomous motion by introducing the feedback loop. In ref. 13, all actuations were achieved by externally heating and cooling the polymer repeatedly, whereas in what follows, we show fast actuations self-sustained on a surface of constant temperature, without external intervention of switching the temperature up and down.

## Results and discussion

Autonomous, continuous motion was indeed achieved (Fig. 1b and c and Movie S1†). To prepare the specimen of EVA used, a strip of initial length 35 mm (4.3 mm in width and 0.9 mm in thickness) was stretched at 90 °C to a length of 75 mm, followed by cooling under a constant strain to room temperature for shape fixation. Prior to the actuation test, the elongated strip was heated on the hot surface of a steel plate (*T*_surf_ = 75 °C) to purposely shrink it to 65 mm (remaining strain 100%, see Methods in ESI†) to release part of the entropic elastic strain energy that, if overpowering the internal stress arising from the volume change associated with melting and crystallization, can cause irreversible contraction. This programming phase resulted in the test specimen denoted as 0.6 mm-100%, *i.e.*, 100% elongation and 0.6 mm in thickness (all specimens were prepared to have the same length of 65 mm and width of 3 mm). It was then placed on the substrate surface (*T*_surf_ = 84 °C) located in a hood with air flow velocity of 160 ± 5 feet per min, and the autonomous actuation was recorded by a camera from which frame-by-frame analysis (photos or data) could be conducted. As is seen in Fig. 1, once in contact with the hot surface, the middle of the strip jumps up in the air to form an arch, which is a clear manifestation of an inward contractile force acting on the bottom layers of the specimen due to the melting of crystallites. Shortly after, the strip went down to flatten on the surface, which indicates an extensional force acting on the same side of the specimen as a result of the recrystallization of the oriented chain segments in the air. This oscillating motion then went on continuously in a self-sustained and self-powered manner, fuelled only by the thermal energy provided by the hot surface. The first two jumps occurred within 2 seconds (Fig. 1b). Although the height of the arch decreased over time, it remained substantial even after 500 cycles over 35 min of actuation (Fig. 1c). Therefore, given a substrate surface as a heat source and air flow above, a thermo-mechano-thermal feedback loop is established that governs the actuation of the polymer from sensing high temperature (melting) to jumping up (motion) to sensing low temperature (crystallization) to falling down (motion). The expected temperature variation of the strip during the jump/descent cycles on the substrate surface was observable with an infrared camera (Fig. S1†). In principle, this autonomous actuation would go on perpetually if the melting/crystallization generates the same superficial contraction/extension force in every cycle. In practice, as will be discussed further on, “imperfections” may prevent this from happening.

**Fig. 2:**
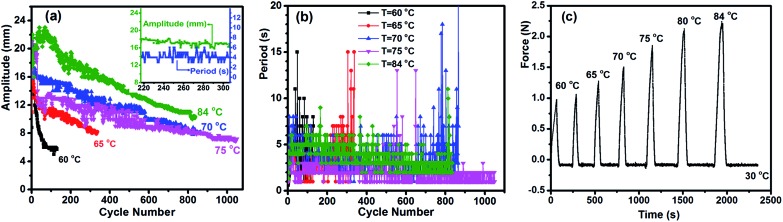
Effect of substrate surface temperature. (a) and (b) Variation of the actuation amplitude (in mm) and period (in seconds), respectively, with the number of jump/descent cycles for the specimen 0.6 mm-100% placed on substrate surfaces set at different temperatures. The inset in (a) (*T*_surf_ = 84 °C) shows the relatively steady state for both amplitude and period over a certain actuation time period. (c) Contractile force for the specimen 0.6 mm-100% heated to different temperatures in the isostrain experiment. Before being heated to a given temperature, the sample under constant strain was cooled to 30 °C.

We investigated the effect of three key parameters on the autonomous actuation by measuring the motion amplitude, defined as the height of elevation, and the period, defined as the time for completing one cycle of jump and descent. They are the substrate surface temperature *T*_surf_, specimen elongation and thickness. Intuitively, both the actuation amplitude and period should be related to the superficial contraction force that pushes the specimen upward at a certain speed. Therefore, to gain more insight into the role played by each variable, we also utilised the isostrain experiment to measure the contraction force developed in the specimen under constant strain while undergoing partial melting of crystallites. Since the jumping sample in air was previously heated during contact with the substrate, its body temperature is higher than that of the air and decreases with time before touching the substrate again. This ensures that the crystallization-induced expansion happens during the non-isothermal process. Although such measured force is generated by the whole sample, the data reveal the effect of a given parameter on the superficial contraction force. First, the substrate surface temperature is obviously important, because it influences the number of crystallites to be melted, which, in turn, determines the contraction force. The results obtained at various *T*_surf_ values show clear trends. Overall, as compared to the relatively low *T*_surf_ of 60 and 65 °C, higher temperatures of 70, 75 and 84 °C led to larger amplitudes during the whole process, longer duration (from 35 to 52 min) and more cycles (from 800 to 1000) (Fig. 2a). However, the larger amplitude apparently resulted in a longer period, which is particularly evident by comparing the data at 84 and 75 °C (Fig. 2b). As for the contraction force, it increased with increasing temperature (Fig. 2c), which is no surprise because a higher temperature increases the number of melted crystallites and thus the proportion of the actuation domains in the specimen. It becomes clear that the actuation amplitude is essentially determined by the contraction force, which, at *T*_surf_ below 60 °C, is not strong enough to drive the actuation. The effect on the period is more complex. Even though a larger amplitude pushes the arch further from the hot surface, a higher *T*_surf_ makes the air above warmer, which may actually slow down the crystallization speed. In the end, the period is mainly affected by the time required for the arch to return to the flat state (Movie S1†), which is determined by how fast the crystallization can generate a sufficient extensional force to drive the motion. Finally, some features of the autonomous actuation can be noticed on closer inspection of the data at 84 °C. At the early stage (within about the first 80 cycles), the amplitude increased to a maximum (23 mm) before going down, displaying large fluctuations. Then, with the amplitude decreasing gradually over time, the actuation appeared to enter a relatively steady state in both amplitude and period, as can be seen from the inset (in Fig. 2a) showing the changes over about 100 cycles. The period decreased with the decreasing amplitude before rising sharply after a total of more than 800 cycles, signalling the end of actuation.

**Fig. 3:**
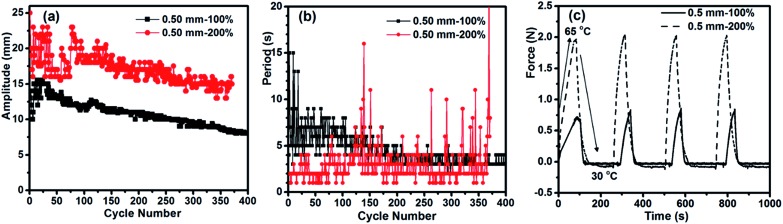
Effect of specimen elongation. (a) and (b) Variation of the actuation amplitude and period, respectively, with the number of jump/descent cycles for two specimens with the same dimensions but different elongation degrees (100% and 200%) (*T*_surf_ = 75 °C). (c) Contractile force variation for the two specimens subjected to several cycles of heating (to 65 °C) and cooling (to 30 °C).

When two specimens of the same dimensions but different elongation degrees were subjected to the autonomous actuation at *T*_surf_ = 75 °C, they behaved differently in both amplitude (Fig. 3a) and period (Fig. 3b). The one with a larger elongation (200%) showed a higher jumping amplitude and shorter period than the one with a smaller elongation (100%). The reason can be found from the contraction force (Fig. 3c). The force generated in the specimen with 200% elongation is much stronger than that in the specimen with 100% elongation. Indeed, the elongation degree determines the amount of strain energy stored in a sample. Upon melting of crystallites in the actuation domains, more strain energy is released from relaxation of more oriented chains in the amorphous region, thus generating a greater contraction force to make the specimen jump faster and to a larger magnitude. For the return to the flat state, since *T*_surf_ is the same, meaning the same temperature gradient in the air above the surface is present, a larger amplitude allows the specimen to crystallize more quickly in the colder air and flattens the arch faster. This explains the shorter period observed for the specimen with 200% elongation. However, the faster autonomous actuation appeared to be accompanied by larger fluctuations in amplitude and a shorter duration. A likely explanation is that at this large elongation, the melting-induced contraction has a more important contribution from the release of entropic elastic strain energy, so that the subsequent crystallization could not induce an elongation that matches the contraction. As the actuation goes on, the extent of irreversible contraction of the lower side of the specimen increases, creating a curvature that, at some point in the process, prevents the specimen from touching the hot surface firmly and basically puts an end to the actuation. Likewise, the specimen thickness also influences the autonomous actuation (see Fig. S2 and the related discussion in the ESI†). Although the achieved hour-long, autonomous actuation self-powered from a hot surface is remarkable, what brings the actuation to an end should be discussed. In principle, to have the oscillating motion go on with a constant amplitude and period, each actuation cycle must proceed under exactly the same melting-induced contractile force, the same crystallization-induced extensional force, as well as the same frictional force between the two ends of the strip and the substrate surface. In practice, however, many factors can intervene and deny the specimen a steady-state motion. We carried out X-ray diffraction and DSC measurements on a specimen before actuation and after reaching the end of actuation lasting over 1000 cycles. No decrease in crystallinity (Fig. S3, Table S1†) and no deterioration of crystallite alignment was observed after actuation (Fig. S4†), implying that the end of actuation was not caused by structural changes in the EVA samples. It seems that the main cause of the decaying autonomous actuation over time is the uneven and weakening contact of the specimen with the hot surface. Generally, as the amplitude decreases, the extensional force that flattens the strip becomes smaller, which results in a “loose” contact between them and, consequently, a less effective heating of EVA and melting of the crystallites. This, in turn, leads to an even smaller jump amplitude that translates into a looser contact after the descent. Such a deteriorating cycle continues to eventually end the actuation. Experimentally, the end of actuation was approached when the specimen experienced a sudden rise in period. Another observation supported this analysis. After an hour-long actuation (over 1000 cycles) was ended, compressing the specimen slightly against the hot surface could reactivate the autonomous actuation for a similar number of jump/descent cycles. With a sample of 0.5 mm-100% (*T*_surf_ = 75 °C), this manual reactivation was repeated several times, resulting in over 8000 cycles totalling 8 h of actuation!

**Fig. 4:**
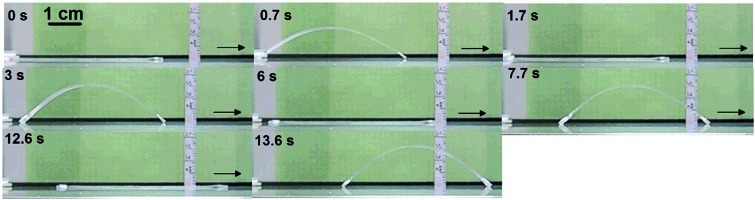
Snapshots showing the self-walking of a specimen (100% elongation, 65 × 3 × 0.5 mm^3^) on the substrate surface (*T*_surf_ = 75 °C). The left end of the specimen was made to have more friction with the substrate than the right end.

**Fig. 5:**
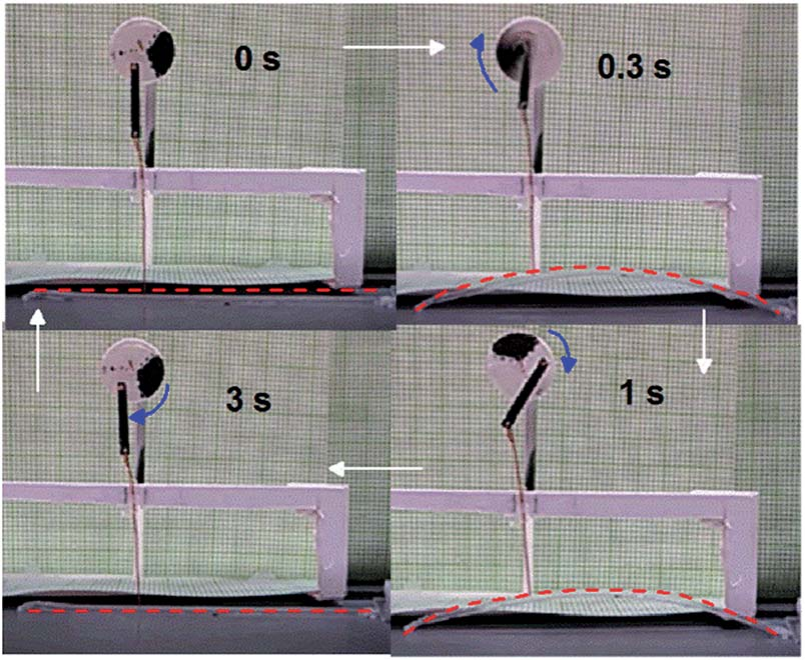
Snapshots showing the rotation of a wheel driven by the autonomous actuation of a specimen (100% elongation, 65 × 3 × 0.9 mm^3^) on the substrate surface (*T*_surf_ = 75 °C). The strip actuator is marked with a red dashed line for clarity.

The autonomous motion of the polymer specimen is self-fuelled from the hot surface. Simple tests could be designed to appreciate the conversion of thermal energy to mechanical work in a self-sustained manner. During the up and down motion, the two ends of the specimen slide on the substrate surface toward and away from each other, respectively, when the friction at the two ends is made similar by controlling the roughness. A low and even friction between the specimen and substrate on both sides facilitates the actuation and helps reach a steady-state of motion. However, with one end of the specimen purposely made rough to increase the friction with the substrate surface, the continuous jump and descent motion could lead to “walking” of the specimen in the direction of the end with lower friction (Fig. 4 and Movie S2†). In another handmade device, one end of a thin rod is attached to the upper side of a specimen and the other end is joined to the arm of a wheel. By putting the specimen on the substrate surface, the up and down actuation could make the wheel rotate 25 times over 1 min (Fig. 5 and Movie S3†), mimicking a diaphragm pump. As a final note, in all autonomous motion tests, despite the fact that the two ends of the specimen were in constant contact with the substrate surface, rigid skeleton domains should remain because *T*_surf_ was lower than *T*_m,high_. Even with all the crystallites melted in the two ends over time, the autonomous motion of the central section of the specimen would continue as long as the thermo-mechano-thermal feedback loop persisted.

## Conclusions

In summary, we have demonstrated a strategy that allows thermal phase transition-based polymer actuators, like crosslinked EVA, that can undergo repeated contraction/elongation upon repeated heating/cooling cycles, to exhibit autonomous, self-sustained motion with no need for temperature switching. We showed that by placing a flat specimen of EVA with aligned crystallites on the surface of a heated substrate, crystallite melting-induced longitudinal contraction occurs on the lower side of the specimen in contact with the substrate, and the unbalanced contraction force elevates the central section of the specimen to form an arch. Once in the air, the specimen is cooled down and oriented chain segments recrystallize, which induces an extensional force on the same side that brings the specimen back to the flat state and, by doing so, causes the specimen to touch the substrate surface to reinitiate the actuation cycle. This way, a thermo-mechano-thermal feedback loop can be created, which is indispensable for the autonomous actuation only fuelled by the hot substrate surface. We investigated the effect of substrate surface temperature, specimen elongation and thickness on the jump amplitude and the period of the oscillating motion. Hour-long actuation with over a thousand cycles of motion was achieved. Such continuous motion of a solid polymer driven by thermal energy and without on/off temperature switching is unprecedented. Based on the validated mechanism, potentially exploitable polymer actuators are not limited to semicrystalline polymers like EVA. Any polymer that displays reversible contraction and elongation associated with a reversible thermal phase transition could be applied for such autonomous actuation. Moreover, the potential of doing mechanical work on a hot substrate subsurface by making use of direct thermomechanical energy conversion was demonstrated.
