# Supplementary material for: A new function for thermal phase transition-based polymer actuators: autonomous motion on a surface of constant temperature
Source: Chem Sci. 2017 Jul 3;8(9):6307–12. doi: 10.1039/c7sc01792h (PMC5628403; doi:10.1039/c7sc01792h)
Supplement: Supplementary file 1 [file SC-008-C7SC01792H-s001.pdf]

Electronic Supplementary Material (ESI) for Chemical Science.
This journal is © The Royal Society of Chemistry 2017

Supporting Information

New Function for Thermal Phase Transition-Based Polymer Actuators: Autonomous Motion on a Surface of Constant Temperature

*Feijie Ge and Yue Zhao**

Materials and Methods

Sample preparation. 6 g EVA (containing 18 wt.% of VA, Sigma-Aldrich, Canada) was first dissolved in 50 mL toluene at 80 °C under magnetic stirring to form homogeneous solution, which was then cooled to 55 °C and subsequently added with 0.123 g DCP (dicumyl peroxide, Sigma-Aldrich, Canada) and continuously stirred for 10 min. The above solution was first cast into a petri dish to allow evaporation of the solvent overnight in the hood, then moved to a vacuum oven and dried at 60 °C for 48 hours to remove the residual solvent. The obtained composite containing 2.0 wt.% DCP was cut into particulates and then hot-compressed (170 °C and 2.5 MPa for 10 min, then 190 °C and 3.0 MPa for 10 min, and finally 200 °C and 3.5 MPa for 25 min) and crosslinked (using Carver Laboratory Press, Model C, Fred S. Carver Inc., USA) in metal molds into sheets with desired dimension.

Programming aligned specimens. Strips were cut off from the above prepared samples, and stretched manually at a rate of about 5 mm s^{-1} (above 90 °C) with tweezers to desired elongations ε_p, ε_p=100%×(L_p-L_0)/(L_0-5 mm), where L_0 is the original length before stretching, L_p is the length after stretching. In the above equation, 5 mm was subtracted since the corresponding parts (with a total length of 5 mm) covered by the tweezers at the two ends were not stretched. For example, a strip with original dimension of 35 mm in length, 4.3 mm in width and 0.7 mm in thickness was stretched to 75 mm with ε_p of 133%, and then heated on the hot surface at 75 °C to reduce the length to 65 mm, with final ε_p of 100%. Then the elongated samples were cooled in air under constant strain at room temperature for shape fixation. For the strips with final elongation of 200%, the original length and width were 2.5 mm and 4.8 mm, respectively. All samples were programmed to the final length of 65 mm and width of 3 mm to perform the tests and measurements.

Tests. The autonomous oscillating motion was characterized by monitoring the amplitude and period for each cycle for a given specimen put on a hot surface made of steel and heated under control by a hot plate (Fisher Scientific, USA). All tests were conducted in a hood (equipped with AFA 1000 airflow monitor, motto manufacturing LTD, England) with velocity of air flow at 160±5 fpm. Both the amplitude and the period were read from the video.

Measurements. The isostrain experiments were performed at a given temperature in a tensile machine (Instron 5965 universal testing system equipped with a chamber oven and controlled by Bluehill 3 software, UK). Samples were clamped and held at constant strain in the chamber, and subjected to heating at 36 °C min^{-1} and cooling at 10 °C min^{-1} while the forces were monitored in real time. The temperature change of the sample during a motion cycle was monitored using an infrared thermometer (FLIR TG165). DSC measurements were carried on a TA Q200 instrument. Samples were heated from 20 °C to 100 °C at a rate of 5 °C min^{-1} under nitrogen flow with a

rate of 50 ml min^{-1}. Data from the first scan were collected for analysis. WAXS and SAXS patterns were collected with a Bruker AXS Nanostar system equipped with a Microfocus Copper Anode at 45 kV / 0.65 mA, MONTAL OPTICS and a VANTEC 2000 2D detector at 90 mm distance (WAXS) and 1070 mm distance (SAXS), respectively, from the samples calibrated with a Silver Behenate standard.

Additional Results and Discussion

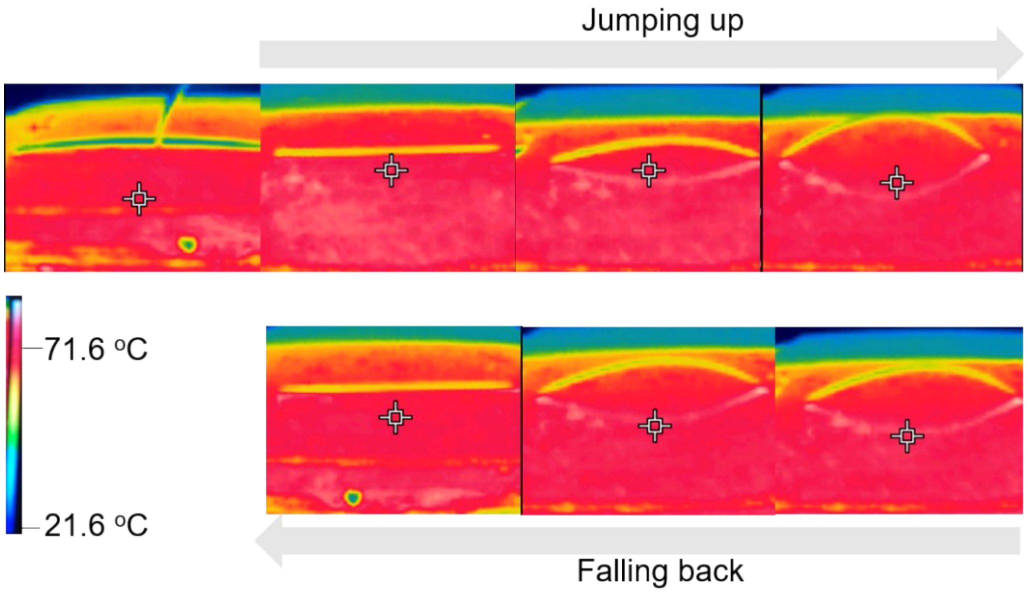

Fig. S1 Infrared images showing the temperature variation in a strip of 0.5 mm-100% during one up/down motion cycle on the substrate surface at T_{surf}=75 ^{o}C. The first image on the left of the upper row was recorded with the strip in the air before being deposited on the hot surface.

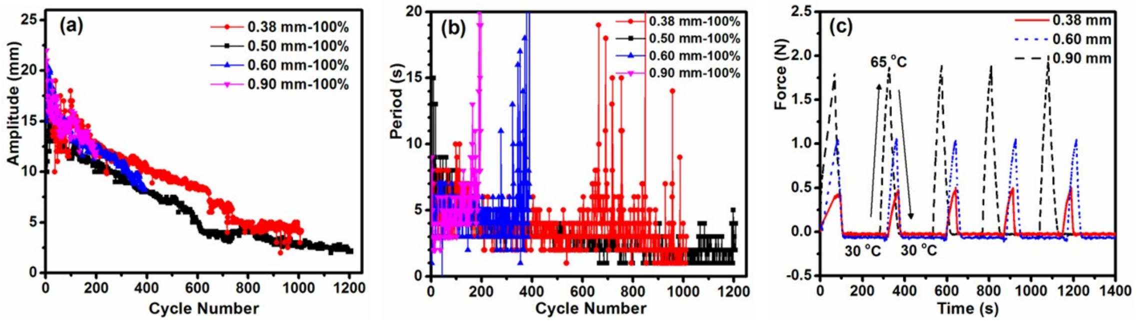

Fig. S2 Effect of specimen thickness. (a) and (b) Variation of actuation amplitude and period, respectively, over the number of jump/descent cycles for specimens of different thicknesses (100% elongation and on substrate surface at T_{surf}=75 ^{o}C). (c) Contractile force variation for three specimens of different thicknesses subjected to several cycles of heating (to 65 ^{o}C) and cooling (to 30 ^{o}C).

To investigate the effect of specimen thickness, four samples were prepared to have the same elongation degree (100%), length and width but different thicknesses ranging from 0.38 to 0.90 mm. The results using T_{surf}=75 ^{o}C show that the thickness had an important effect on the number of completed cycles, i.e., the total actuation time (Fig. S2a and S2b). While the thicker ones stopped jumping earlier in time with fewer cycles, the two thinner

samples could autonomously actuate much longer, over 1 h. As expected, the contraction force generated by the whole volume of specimen increased with increasing the thickness (Fig. S2c), which, however, is not telling about the contraction force responsible for the jumping up and down actuation. At the same T_{surf}, the amount of melted crystallites would be similar in all specimens regardless of the thickness, which means a larger fraction of melted crystallites and thus larger proportion of actuation domains for a thinner specimen. This appears to be necessary for a sustainable, self-powered actuation. From the above, it appears certain that the performance of the autonomous actuation, in terms of amplitude, speed and duration, is affected by the interplay of a number of parameters. Although a precise and complete control on the actuation seems difficult at this point, the key role of some parameters is clear. On the one hand, the jump amplitude is mostly governed by the melting-induced contraction force. At a given specimen thickness, bigger contraction force can be obtained by using either higher T_{surf} to increase the fraction of melted crystallites, or higher elongation degree to store more strain energy in the specimen. On the other hand, the period is mainly determined by the time required for the specimen to flatten from the arch shape. Large extensional force from fast crystallization would induce fast actuation with short period. However, what induces a large contraction force upon crystallite melting cannot ensure fast actuation, which is the case of using high T_{surf} that could warm the air around the specimen and thus slow down the crystallization.

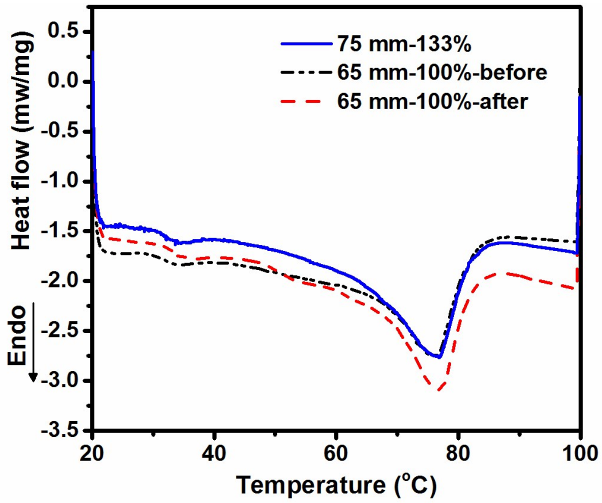

Fig. S3 DSC (first heating) curves for samples preprogrammed (blue solid), before jumping (black dash dot dot) and after jumping (red dash).

Table S1. Thermal parameters of EVA samples obtained from DSC measurement.

Sample	T_m [°C][a)]	ΔH_m [J g^{-1}] [b)]	X_c [%][c)]
75 mm-133%	76.8	-46.43	16.8
65 mm-100%-before	76.5	-45.31	16.4
65 mm-100%-after	76.8	-48.73	17.6

[a)] T_m is the peak melting temperature during the first heating cycle. [b)] ΔH_m is the enthalpy of melting. [c)] X_c is the crystallinity obtained from the first heating using $X_c = (\Delta H_m/\Delta H^*_m) \times 100\%$, where ΔH^*_m is the enthalpy of melting of perfect polyethylene crystal, 277.1 J g^{-1} based on reference[1].

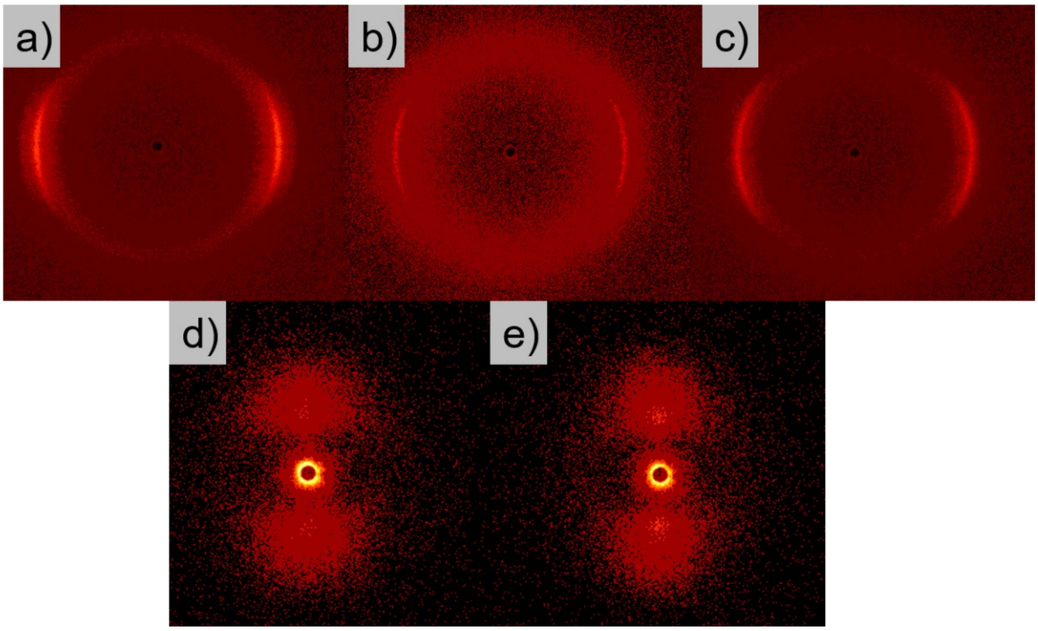

Fig. S4 2D-WAXS patterns (upper series) for preprogrammed sample 75 mm-133% (a), sample 65 mm-100% before jumping (b), after jumping for 1000 cycles (c) and 2D-SAXS patterns (lower series) for sample 65 mm-100% before jumping (d) and after jumping for 8000 cycles (e).

Supporting References

[1] R.P. Quirk and M. A. A. Alsamarraie in *Polymer handbook*, *3rd edition* (Eds.: J. Brandrup, E. H. Immergut), Wiley-Interscience Publication, New York, **1989,** pp. V/19.
